# Supplementary material for: Conditional chemoconnectomics (cCCTomics) as a strategy for efficient and conditional targeting of chemical transmission
Source: eLife. 2024 Apr 30;12:RP91927. doi: 10.7554/eLife.91927 (PMC11060718; doi:10.7554/eLife.91927)
Supplement: Figure 3—source data 3. [file elife-91927-fig3-data3.zip › Figure 3 source data3 part 1.zip!/Figure 3-Source data 3-part1/1067-9-CRISPResso_Report_c33kf3u3.zip!/CRISPResso_on_1067_12/1a.Read_barplot.pdf]

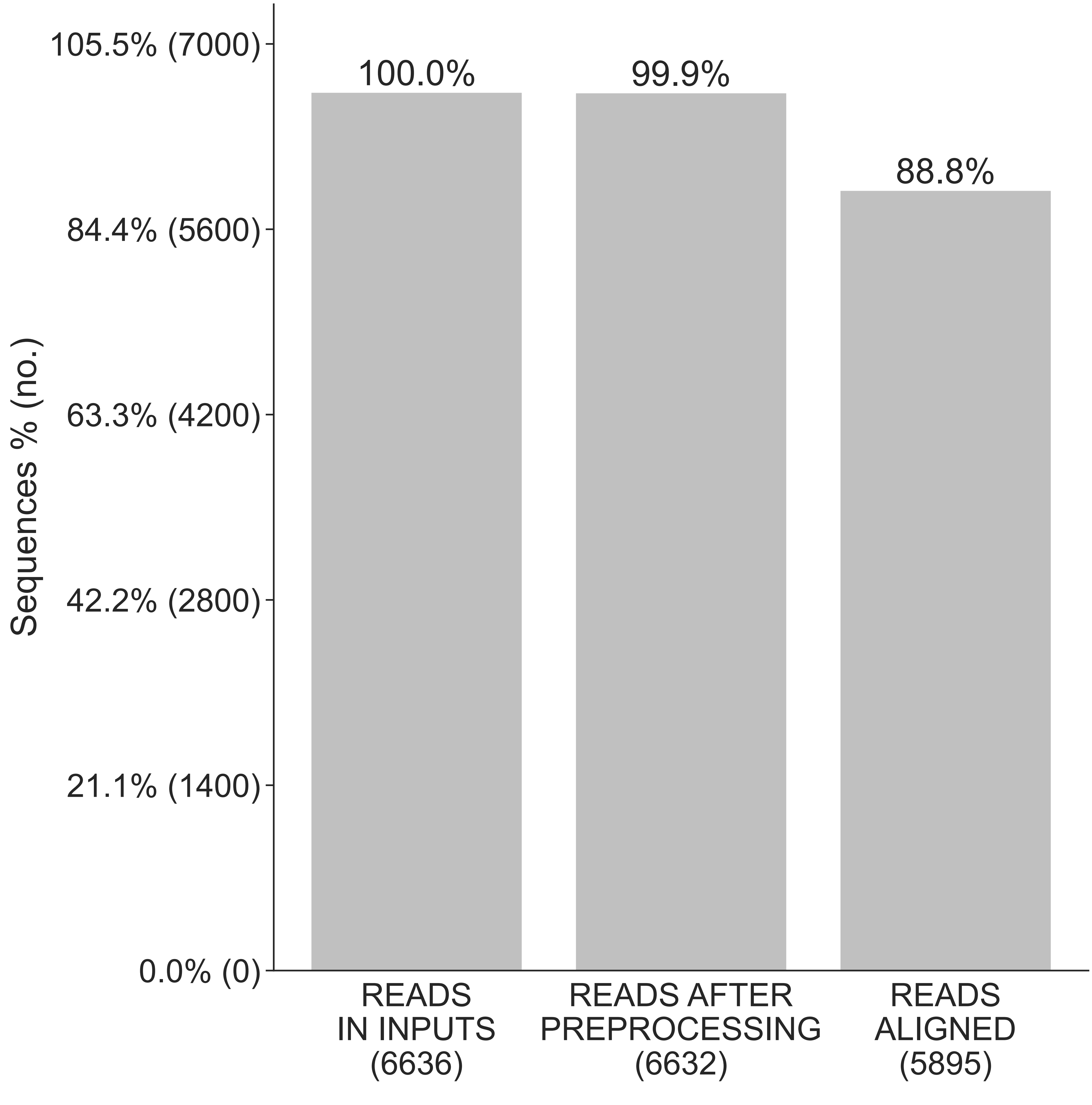

Sequences % (no.)
105.5% (7000)
84.4% (5600)
63.3% (4200)
42.2% (2800)
21.1% (1400)
0.0% (0)
100.0%
99.9%
88.8%
READS
IN INPUTS
(6636)
READS AFTER
PREPROCESSING
(6632)
READS
ALIGNED
(5895)
